# Gutted: constipation in children with chronic kidney disease and on dialysis

**DOI:** 10.1007/s00467-022-05849-y

**Published:** 2023-01-09

**Authors:** Mandy Wan, Lillian King, Natasha Baugh, Zainab Arslan, Evelien Snauwaert, Fabio Paglialonga, Rukshana Shroff

**Affiliations:** 1 Evelina Pharmacy Department, Evelina London Children’s Hospital, NHS Foundation Trust, Guy’s and St ThomasWestminster Bridge Road, London, SE1 7EH UK; 2 Institute of Pharmaceutical Science, King’s College London, London, UK; 3 UCL Great Ormond Street Hospital for Children and Institute of Child Health, London, UK; 4 Department of Nephrology, Ghent University Hospital, Ghent, Belgium; 5 Fondazione IRCCS Ca’ Granda Ospedale Maggiore Policlinico, Milan, Italy

**Keywords:** Constipation, Laxative, Gut microbiota, Chronic kidney disease, Dialysis

## Abstract

Functional constipation is a common problem in otherwise healthy children. Children with chronic kidney disease (CKD) and on dialysis have additional disease-related risk factors including the uremic milieu, fluid and dietary restrictions, and decreased physical activity, as well as treatment-related risk factors such as dialysis therapy and polypharmacy that contribute to and compound the problem. Constipation causes significant distress for children and their caregivers. In children on peritoneal dialysis, severe constipation can impede catheter function and ultrafiltration. Accumulating evidence points to a possible bidirectional relationship between constipation and CKD, potentially mediated by gut dysbiosis with consequent increased generation of gut-derived uremic toxins and disruption of intestinal epithelium integrity leading to translocation of noxious luminal contents into the circulation inducing systemic inflammation. Effective management of constipation is required but there is little published data on the safety and effectiveness of treatments in adults or children with CKD. In this review, we discuss the diagnosis and epidemiology of functional constipation, provide an overview of its pathophysiology, summarize the therapeutic management, and reflect on the challenges in children with CKD.

**Supplementary Information:**

The online version contains supplementary material available at 10.1007/s00467-022-05849-y.

## Introduction

Children with chronic kidney disease (CKD) experience a multitude of symptoms of which constipation is a frequently encountered clinical problem. Constipation can markedly affect the quality of life and psychological well-being of the child and the family, and it imposes a considerable social and economic burden [1]. Moreover, in patients on dialysis, particularly those on peritoneal dialysis (PD), constipation can displace the PD catheter tip out of the pelvis and impede ultrafiltration, and is by far the most common cause of catheter dysfunction in this cohort [2].

In recent years, there has been a growing interest in understanding the association between gastrointestinal health and chronic diseases, in particular the broader adverse clinical impact of constipation beyond what was once perceived as an innocuous condition [3, 4]. Epidemiological studies have reported an independent association between constipation and cardiovascular disease [4, 5], with several cohort studies showing increased mortality among adults who experience constipation [3, 6]. Meanwhile, data from a nationwide cohort study of veterans, with estimated glomerular filtration rate (eGFR) ≥ 60 mL/min per 1.73 m^2^, demonstrated that individuals with constipation had a significantly higher risk of incident CKD and kidney failure [6]. In the same study, those with constipation showed a greater risk of experiencing more progressive decline in eGFR that was independent of known risk factors [6]. It is of note that a higher overall mortality risk has also been reported in association with laxative use in hemodialysis patients, where deaths from infection and cancer were significantly associated with laxative prescription [7]. These findings suggest that constipation could play a potential role in the progression of kidney failure and its complications, making the case for a proactive approach to its clinical management in patients with CKD.

Yet, constipation is not well studied in the CKD population, and the evidence base remains limited even for pharmacological interventions that are widely used in clinical practice. The scarcity of studies in adults was highlighted by the Kidney Disease: Improving Global Outcomes controversies conference on supportive care where the scarcity of evidence in children precluded its inclusion in the review [8]. The lack of evidence and management guidelines thus leave clinicians to rely on their subjective judgement when making treatment decisions. In this context, this review aims to provide an overview of the current understanding of the pathophysiology of functional constipation in children, to summarize the intrinsic effects and extrinsic factors related to CKD leading to gastrointestinal changes, and to discuss the therapeutic management of and challenges in children with CKD.

## Diagnosis

Constipation has traditionally been classified as either organic or functional. In children, organic causes are uncommon, and the etiology varies from Hirschsprung disease, anorectal malformations, neuromuscular disease, and endocrine disorders [9]. Constipation may also be secondary to metabolic disturbances and medication use [9]. The presence of any physiological or anatomical abnormalities thus requires appropriate medical evaluation before diagnosis of functional constipation.

Functional constipation is constipation without evidence of an organic cause; it is among the spectrum of functional gastrointestinal disorders, also known as disorders of gut-brain interaction [10–12]. These disorders are characterized in terms of cluster of symptoms, and their pathophysiology relates to any combination of motility disturbance, visceral hypersensitivity, altered mucosal and immune function, altered gut microbiota, and altered central nervous system processing [10]. The symptoms of functional constipation are typically characterized by infrequent bowel movements, painful defecation, difficult passage of hard stools, and/or sensation of incomplete evacuation of stool, where constipation can also lead to overflow fecal incontinence in some patients. In 95% of children presenting with symptoms, cases are considered to be of functional origin [9]. However, this dichotomous classification is overly simplistic, particularly in the setting of CKD where the pathophysiology is often multifactorial.

In clinical practice, the diagnosis of functional constipation is based on a thorough medical history and physical examination [11]. Additional testing, such as rectal ultrasound, transit studies, and abdominal radiograph, are generally not recommended in the absence of specific signs and symptoms [10–12]. In some cases, such as in children with bladder bowel dysfunction, ultrasonic measurement of the transverse rectal diameter during routine abdominal ultrasound may be helpful to support the diagnosis of constipation [13]. The two most commonly used diagnostic tools for functional constipation are the Rome criteria and the Bristol Stool Form Scale (BSFS) [10, 11, 14], with the BSFS being more commonly used to characterize acute changes in bowel habits in hospitalized patients. The Rome criteria is a symptom-based criteria for diagnosing the presence of functional constipation (Supplementary Table 1) [10]. The BSFS (Supplementary Table 2) is a 7-level scale based on visual inspection of feces with respect to texture and morphology, and provides a useful indicator of gastrointestinal transit time [14].

## Epidemiology

The prevalence of constipation in patients with CKD shows substantial variation across studies, which in part reflects differences in diagnostic criteria and variations in assessment of bowel habits on history taking. A systematic review of 30 studies, with a total of 5161 adults on dialysis, reported a prevalence ranging from 1.6 to 71.7% and 14.2 to 90.3% in hemodialysis (HD) and PD patients, respectively [15]. Furthermore, studies that have compared the prevalence between HD and PD patients reported an increased risk of constipation in those on HD [3]. In a cross-sectional study which included 478 HD and 127 PD patients, patients on HD had a 4.2 times increased risk of constipation compared to those on PD [16]. A 3.1 times higher risk in HD patients was similarly reported by Yasuda et al. [17]. Using radiopaque markers, the study by Wu et al. also demonstrated that both segmental and total colonic transit times were significantly longer in HD than in PD patients and healthy controls, with respective mean total colonic transit times of 43.0, 32.7, and 24.3 h [18].

Data from patients with non-dialysis-dependent CKD are scarce. Ramos et al. reported a prevalence rate of 34.9% (based on Rome III criteria) and 32.6% (based on the BSFS) in a study of 43 adult patients with eGFR of 21.3 ± 7.9 mL/min/1.73 m^2^ [19]. In another small cross-sectional study including 21 patients with non-dialysis-dependent CKD (eGFR < 15 mL/min/1.73 m^2^), the prevalence was reported to be 4.8% and 19.0% based on the Rome III criteria and the BSFS, respectively [20]. There are no published data reporting on the prevalence of constipation in children with non-dialysis or dialysis-dependent CKD.

This review focuses on children with CKD, but a similar situation is seen in individuals with polyuric kidney diseases who typically have chronic constipation. The treatment plans are very similar as discussed below.

## Pathophysiology

**Fig. 1:**
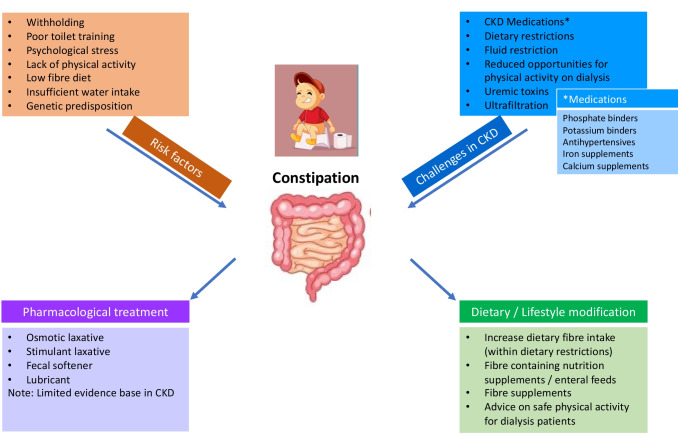
Constipation in children with chronic kidney disease and its management strategies

The pathogenesis and pathophysiology of constipation, even in the general population, is poorly understood and considered to be multifactorial (Fig. 1). In the general pediatric population, contributing factors include withholding, poor toilet training, psychological stress, lack of physical activity, low-fiber diet, inadequate water intake, and genetic predisposition.

The physiology of defecation and maintenance of continence is a coordinated process requiring rectal filling, awareness of rectal filling, and the ability to evacuate the stool and control the pelvic floor muscles. This fundamental physiological process requires the coordination and integration of neural, muscular, endocrine, immune, and cognitive systems, and disturbances in any of these systems can cause constipation.

In children, an initial painful episode of bowel movement leads to voluntary withholding behavior due to fear of pain, which causes the child to enter a vicious cycle of withholding and worsening constipation [9]. The prolonged stasis and accumulation of fecal mass leads to increased mucosal fluid absorption from the retained stools which become progressively more difficult to evacuate [9]. As the cycle is repeated, the rectum becomes dilated, leading to a decrease in anal muscle contraction efficiency and loss of response to a sense of defecation urge. Psychological factors, through the brain-gut axis, are also thought to modulate colonic and rectal functions.

## Pathophysiology in the setting of CKD

Additional disease-related risk factors are likely to be implicated in the underlying pathophysiologic disturbances of constipation in children with CKD (Fig. 1). Several inter-related mechanisms have been proposed, including impaired gastrointestinal motility and barrier function, dysregulation of the brain-gut-kidney axis, inflammation, and alterations in the gut microbiome.

### Uremia-related changes

As kidney function declines, the progressive electrolyte imbalance, uremia-related biochemical changes, and increased concentrations of oxidative stress biomarkers have been suggested to play an important role. A recent experimental study by Nishiyama et al. showed a decreased amount of stool in a 5/6 nephrectomized mice CKD model, and constipation was correlated with a suppressed contraction response in ileum motility and decreased relaxation response in distal colon motility [21]. Similarly, Hoibian et al. showed a 1.8-fold longer gastrointestinal transit time in adenine-induced CKD mice as compared to control, and when the resected colons from control mice were then incubated with healthy and uremic plasma, those incubated with the latter exhibited a blunted level of contraction [22]. This proposed uremic toxin mechanism is further supported by clinical data where increased concentrations of uremic toxins such as p-cresyl sulfate and indoxyl sulfate have been reported in both dialysis- and non-dialysis-dependent CKD patients with constipation [19, 23].

Meanwhile, studies have shown that impaired intestinal permeability could constitute an important part of the pathogenic mechanism. It has been hypothesized from experimental studies that the uremic milieu impairs intestinal permeability by reducing the protein expression of tight junction proteins, leading to translocation of microorganisms and their components into the systematic circulation driving a pro-inflammatory immune response [24]. While data in humans are limited, changes in the intestinal barrier have been reported in colonic biopsies of CKD patients and are further reflected in results from studies demonstrating endotoxemia, thus suggestive of increased intestinal permeability, across the spectrum of CKD [24, 25]. Direct evidence for an impaired intestinal barrier to causally result in constipation has not been established. Nonetheless, there is converging evidence and a strong theoretical basis that point to a prominent role of inflammation in accelerating kidney disease progression that in turn leads to progressive accumulation of uremic toxins, thereby further affecting intestinal motility [24, 26].

### Medications

Medications commonly used to treat CKD are known to cause constipation. These include phosphate binders, potassium-lowering agents (e.g., calcium resonium), calcium channel blockers (e.g., amlodipine), aluminum-containing drugs (e.g., sucralfate and antacids), and iron supplements. It is of note that the concomitant use of these medicines increases with progression of CKD, but at present, the impact of the cumulative risk is unclear.

Of the different phosphate binders, sevelamer carbonate is reported to be associated with the highest risk; in adults, sevelamer increased constipation compared with calcium, lanthanum, and iron-based phosphate binders with an odds ratio of 2.12, 3.03, and 3.15, respectively [27]. Sevelamer binds bile acids, and since bile acids increase colonic secretion of water and electrolytes and stimulate propagating colonic contractions, sevelamer-induced constipation is partly thought to be attributed to its bile acid adsorption properties [26].

Iron supplement is also a well-established iatrogenic cause of constipation [28]. The mechanism by which it induces constipation is less clear, but it is thought that it may involve oxidative stress and changes to the gut microbiome [28]. Another risk factor for drug-induced constipation in CKD patients is vitamin D therapy. Although the side effect of constipation is rarely seen with vitamin D therapy, constipation is a symptom of hypercalcemia which may be attributable to chronic use of high-dose vitamin D therapy particularly with co-administration of calcium-based phosphate binders.

### Fluid and dietary restriction

The majority of patients with advanced CKD have oligo-anuric kidney failure necessitating strict fluid restriction. Also, patients with advanced CKD are generally recommended a low potassium intake to minimize the risk of hyperkalemia. As a consequence, this typically lowers the intake of dietary fiber as consumption of vegetables, fruits, and whole grains, which are often high in potassium, are reduced. Together with fluid restriction to avoid volume overload and adherence to a kidney diet, these go against the general lifestyle advice for the prevention of constipation.

Studies quantifying the consumption of dietary fiber in the pediatric CKD population are scarce [29], but the available data point to similarly low intakes as seen in the general population. In the general population, it has been reported that intake of dietary fibers is consistently lower than recommended targets across all age groups in all regions of the world [30]. In the UK, data from the National Diet and Nutrition Survey rolling program (2016–2019), a cross-sectional survey of the general UK population, reported mean daily fiber intake of between 10.4 and 16 g in children [31]. These quantities are significantly lower than government recommendations, with only 12% of 1.5- to 3-year-olds, 14% of 4- to 10-year-olds, and 4% of 11- to 18-year-olds, meeting the recommended dietary requirements [31]. In a cohort of 61 non-dialysis children, El Amouri et al. showed that only 39% of children with CKD 1–3 and 9% of children with CKD 4–5 met the fiber intake recommendations, with three patients receiving no dietary fiber intake as a result of being exclusively fed by a powdered amino acid formula or a nutritional formula adapted to the needs of CKD [29].

### Decreased physical activity

Research into physical activity in the pediatric CKD population is limited, but available evidence consistently suggests that children with CKD have deceased physical activity compared to the general pediatric population [32]. Physical inactivity is seen even in the early stages of CKD and progressively worsens as the disease burden increases [32]. A number of factors, including the accumulation of uremic toxins, anemia, and reduced cardiac function, have been attributed to increased fatigue, lower muscle strength, and reduced cardiorespiratory fitness, thus resulting in reduced physical activity [32]. In those on dialysis, participation in physical activity is further restricted by the significant amount of time they spend attached to a dialysis machine.

The extent to which physical inactivity is associated with constipation is, however, unclear and likely reflects the methodological challenges in conducting studies on this topic [33]. A recent meta-analysis in adults on HD reports that exercise therapy may be an effective treatment option for patients with constipation [34].

## Gut dysbiosis

The gut microbiome is an ecosystem of trillions of different microorganisms and their collective genomes. These microbes form a very complex dynamic and symbiotic ecological entity that is in constant interaction with the host and plays a key role in immune system regulation, intestinal integrity, amino acid homeostasis, and production of vitamins, as well as being responsible for essential metabolic pathways. Accumulating evidence underlines the potential involvement of these microbial communities in the pathophysiology of constipation. Moreover, increasing attention is also being given to the independent associations between gut microbiota dysbiosis, defined as imbalances in the composition and function of these intestinal microbes, and many diseases ranging from cardiovascular disorders to cancer, and CKD [3].

### Gut dysbiosis in constipation

In an earlier study using culture-based analyses of fecal samples, Zoppi et al. reported that constipation in children was associated with increased abundance in *Clostridium* and *Bifidobacterium* species compared to healthy controls [35]. Using quantitative real-time polymerase chain reaction, Jomehzadeh et al. showed that constipated children had lower levels of *Lactobacillus* spp. than controls [36]. Several other studies in children with constipation have similarly reported ecological differences of the gut microbiome, but results to date have not provided consensus on the gut microbial characteristic [37, 38]. While some of the differences may be attributable to differences in analysis techniques, and there are limitations in that some studies did not take account of dietary adherence, inconsistent observations to some extent have likewise been reported in adult studies [39]. It remains unclear which gut microbiota signatures are associated with constipation, nor do the data necessarily imply a causal role for gut dysbiosis in constipation, but it is conceivable that the co-presence of both constipation and an altered gastrointestinal environment can further drive the pathophysiology of constipation where prolonged colonic transit time encourages the amplification and colonization of slow-growing species, which in turn alters the microbial ecology of the colon.

Several experimental studies have investigated the potential molecular mechanisms by which gut microbiota dysbiosis can modulate gut functions. Current evidence points to various signal pathways mediated by the metabolites of bacterial fermentation, mediators released by the gut immune response, or intestinal neuroendocrine factors, of which short-chain fatty acids, bile salts, methane, and 5-hydroxytryptamine have been reported to play a role [40]. While their precise mechanisms remain to be elucidated, it would be necessary for future studies to take account of the opposing effects of these factors on intestinal motility, secretion, and colonic fluid transport as well as sensory transmission to assess the overall clinical significance.

### Gut dysbiosis in CKD

The impact of the gut microbiome in CKD and its associated complications has been the focus of increasing research, with several studies investigating links between dysbiosis and pediatric CKD. Hsu et al. performed the largest pediatric study to date in a cohort of 115 children and adolescents with CDK stages 1–4 [41]. The researchers found that plasma concentrations of gut microbiota-dependent methylamines (dimethylamine, trimethylamine, and trimethylamine N-oxide), which have been linked to CKD and cardiovascular disease, were higher in children with CKD stages 2–4 vs. CKD stage 1, and that plasma dimethylamine and trimethylamine concentrations were inversely associated with high blood pressure load as well as lower eGFR [41]. Also, children with CKD who had normal or abnormal blood pressure displayed two distinct microbiota profiles, with the latter showing decreased counts of certain bacteria populations [41]. The authors concluded that gut microbiota-dependent methylamines are related to BP abnormalities and CV risk in children with CKD and postulated that the decrease in bacteria populations had beneficial cardiovascular effects [41]. The findings from this study build on earlier work which have similarly reported changes in the gut microbiota or metabolites in a uremic milieu [42], although the type of dysbiosis and its impact on the pathogenesis of CKD require further investigation.

Data generated from studies in adult CKD patients provide further mechanistic insights on the gut-kidney axis. Both adults and pediatric CKD patients exhibit evidence of a dysbiosis associated with declining kidney function, with changes reported as early as stage 2 [41, 43]. An altered species composition and lower microbial diversity in the gut microbiota have also been reported in kidney transplant recipients [44]. While further research is required to decipher the differences in the gut microbiota and metabolites among patients with different stages of CKD, there is a general picture emerging in which CKD-associated dysbiosis is characterized by an expansion of urease, uricase, and indole- and p-cresol-forming enzymes and a contraction of bacterial families producing beneficial short-chain fatty acids and consequently a shift from saccharolytic to a proteolytic bacterial fermentation [26].

It has been proposed that the accumulation of uremic metabolites in CKD patients is due to both diminished kidney excretion and increased intestinal generation from altered colonic microbial metabolism [45, 46]. Indeed, Poesen et al. demonstrated that both eGFR and intestinal generation independently determined serum concentrations of p-cresyl sulfate and indoxyl sulfate, the two most studied uremic toxins that are by-products of proteolytic bacterial fermentation of amino acids in the intestine, and whose serum concentrations progressively increase as GFR declines [46]. It is this change in the gut microbiome and its metabolic products that is proposed to contribute to increases in colonic pH and intestinal barrier dysfunction, leading to bacterial translocation and endotoxemia that underpin the pathophysiology of systemic inflammation and cardiovascular disease commonly seen in patients with CKD [45, 47].

To facilitate better understanding of altered intestinal microbiota in CKD, experimental studies were used to isolate the effect of kidney failure from confounding factors. Again, these studies showed substantially lower species richness and differences in fecal metabolite profiles in 5/6th nephrectomy rat models as compared to controls [46, 48]. Moreover, a more detailed analysis revealed marked reductions in tight junction protein abundance of claudin-1, occludin, and ZO-1 in colonic mucosa in these animal models. These findings strongly point to an important and independent influence of reduced kidney function on dysbiosis, but it should be recognized that there is much to learn about the impact of CKD on the gut microbiome, with emerging evidence suggesting that CKD-related factors such as diet may play a more determinant role, possibly outweighing the influence of reduced GFR per se [46].

Accordingly, gut dysbiosis is associated with both constipation and CKD. Given that the prevalence of constipation increases as kidney function declines, it is possible that the presence of constipation further exacerbates gut dysbiosis-related changes, which in turn drive the progression of CKD and its complications, including constipation itself, and therefore perpetuating this vicious circle of disturbance in a bidirectional manner.

## Constipation and CKD

### Peritoneal dialysis

While the prevalence of constipation is reported to be lower in patients receiving PD than HD, the topic of constipation is more widely recognized and discussed in the PD setting. Constipation could directly affect the mechanical properties of the PD techniques and increase the risk of peritonitis. Excessive fecal loading can impair the flow of dialysis fluid and impede the drainage through the PD catheter, thereby reducing ultrafiltration. Severe constipation can displace the PD catheter out of the pelvis into the upper abdomen; in some cases, aggressive treatment of the constipation restores the catheter position, but surgical catheter replacement is required in others. In extreme cases, bacterial translocation across the stretched and friable intestinal wall can lead to enteric peritonitis [49]. In a prospective observational study that evaluated gastrointestinal symptoms in PD patients, constipation was one of the strongest predictors of peritonitis [50].

### Hyperkalemia

Constipation may also be contributing to the pathogenesis of CKD through its implication in potassium homeostasis. The kidney is primarily responsible for potassium homeostasis, with only approximately 10% of the ingested potassium excreted via the gastrointestinal tract. While intestinal potassium excretion is minimal in individuals with normal kidney function, it becomes progressively more important in those with kidney disease. The progressive loss of functional nephrons mediates the adaptive response of increasing potassium secretion in remaining nephrons, along with an increase in intestinal excretion such that studies have shown intestinal potassium excretion reaching approximately 80% of dietary potassium for some patients on dialysis. Therefore, it has been suggested that the coexistence of constipation could counterpoise this important adaptive mechanism for maintaining potassium homeostasis in patients with CKD [3].

Among 13 patients on hemodialysis, increasing the number of stools from one to two to four per day with laxatives significantly lowered mean interdialytic potassium concentration (from 5.9 ± 0.2 to 5.5 ± 0.2 mmol/L) [51]. Evidence supporting this association between laxative use and lower risk of hyperkalemia has been shown in a large cohort study of veterans by Sumida et al., where time-varying laxative use was independently associated with lower risk (adjusted odds ratio = 0.79; 95% confidence interval = 0.76 to 0.84) of hyperkalemia (serum potassium greater than 5.5 mEq/L) during the year preceding kidney failure [52]. These findings raise some important questions about the putative role of constipation in dyskalemia and its negative consequences on cardiovascular outcomes in the setting of CKD. It is thus conceivable that obstruction of the colon could lead to reduced potassium excretion. At the same time, the possibility of increased intestinal potassium absorption needs to be considered due to the slow intestinal transit time and impaction of feces with high potassium content [52]. The concept of using laxatives for the management of hyperkalemia in patients with CKD requires further investigation.

## Clinical management

The effective clinical management of constipation requires clinicians to support patients and their caregivers in the early recognition of the signs and symptoms given younger children often do not report accurate symptoms while older children may be too embarrassed to seek advice. Treatment begins once a working diagnosis of functional constipation is made, and other underlying medical conditions have been excluded. Maintenance treatment may take several weeks until regular bowel habits are established, and most children require ongoing laxative therapy while they remain on dialysis [12]. In children starting on PD, a detailed bowel history should be taken to support proactive treatment of constipation prior to PD catheter insertion. Given that constipation can affect the mechanical properties of the PD techniques, passing at least two stools per day is generally advised to reduce the risk of constipation and PD catheter malfunction.

In this section, evidence on both non-pharmacologic and pharmacologic treatment is presented. It should be of note that high-quality evidence to support clinical management of constipation in children is surprisingly limited [11]. The evidence base is even more limited in the CKD setting where even data from observational studies is sparse [53, 54]. To that end, recommendations for the general population are described and discussed in the context of CKD.

### Non-pharmacological management

#### Diet and lifestyle

Gradually increasing dietary fiber, fluid intake, and physical activity levels are recommended as initial management strategies for adults with constipation [55]. The World Gastroenterology Organisation also indicates that psyllium may be appropriate as a fiber supplement in the management of chronic constipation in adults [55]. However, guidance for children differs from that for adults in that dietary and lifestyle intervention alone are not recommended as first-line treatment [11, 12]. In children with functional constipation, the North American Society for Pediatric Gastroenterology, Hepatology, and Nutrition (NASPGHAN) and the European Society for Paediatric Gastroenterology, Hepatology, and Nutrition (ESPAGHN) joint guidance, and similarly the UK National Institute for Health and Care Excellence (NICE), recommend pharmacological treatment in conjunction with a balanced diet with sufficient fiber, adequate fluid intake, and regular physical activity [11, 12]. With respect to the latter, there are no randomized studies evaluating the effect of increased physical activity in childhood constipation [11, 56], and none has examined the effects in the setting of CKD. However, given the adverse physiological, psychological, and social effects of physical inactivity, encouraging children to participate in regular physical activities, adapted to the needs and goals of the individual, could improve muscle function, exercise capacity, and quality of life in children with CKD [57].

#### Fiber

Dietary fiber has varying definitions and multiple subcategories, but they are essentially non-digestible carbohydrates that have a degree of polymerization of three or more monomeric units, plus lignin, and are not hydrolyzed by the endogenous enzymes in the small intestine of humans [58].

Although traditionally defined by its solubility, recent guidelines propose defining fiber by its physiological effects such as fermentability and viscosity [55, 59]. Insoluble (poorly fermentable/bulking) fibers such as cellulose, wheat bran, and lignin chiefly exert their effects on colonic functions such as stool bulking and shortened transit time while soluble (fermentable) fiber (such as pectin and beta glucans) can soften stool as well as exert beneficial prebiotic effects. Food sources of insoluble (poorly fermentable/bulking) fiber include whole grain cereals, nuts and seeds, and fruits and vegetables (particularly the skins and seeds). Food sources of soluble (fermentable) fiber include fruits, vegetables, oats, and pulses (beans, lentils, chickpeas).

There is considerable variation in the recommended dietary fiber intake for children across different nutritional and government bodies [30], and there are no recommendations for children with CKD. Concerns have been raised that a high-fiber diet in childhood may lead to a feeling of fullness, compromising energy intake, but this is not reported in healthy children [8, 16]. A high-fiber intake may include significantly more potassium [60], and when children with CKD are advised to reduce dietary potassium and phosphate intake, this imposed limitation of fruits, vegetables, legumes, nuts and seeds, and whole grains is likely to inadvertently reduce dietary fiber intake. However, more recently, emphasis has been placed on plant-based diets [61]. The bioavailability of potassium from unprocessed plant food is estimated at no more than 60%, so the practice of avoiding fruits and vegetables from the diet based simply on their potassium content should be discouraged [61]. Furthermore, the higher dietary fiber content of some plants together with their high alkali content may counteract the hyperkalemia-inducing effect of a high-potassium intake [61].

In addition, for high-fiber diets to be effective at preventing and improving constipation, adequate fluid must be taken, and this may not be possible for oligo-anuric children with CKD. A qualified dietitian can provide individualized dietary advice including counseling on appropriate food swaps, education on label reading, and advice on food preparation and cooking methods, to reduce potassium content if appropriate, to facilitate the safe increase in dietary fiber intake while controlling biochemistry in children with CKD. Dietitians would also be able to provide advice on the suitability of commencing powdered and/or liquid fiber supplements into children’s oral diets or enteral feed preparations as well as assessing the appropriateness of fiber-containing nutritional supplements.

#### Probiotics, prebiotics, and synbiotics

The role of probiotics, prebiotics, and synbiotics in the treatment of functional constipation is increasingly being investigated given growing evidence of the association between constipation and gut microbiota dysbiosis. Probiotics are live microorganisms that, when administered in adequate amounts, confer a health benefit on the host. Prebiotics are defined as non-digestible food ingredients that promote the metabolism and proliferation of beneficial bacteria in the host. Synbiotics are a combination of probiotics and prebiotics.

It is hypothesized that administration of probiotics might modify the composition of the gut microbiota, thereby leading to functional changes through influence on colonic motility, water and electrolyte secretion and absorption, lactate and short‐chain fatty acid production, and intraluminal pH. Currently, published data are conflicting, and the clinical efficacy of probiotics in alleviating constipation remains controversial. Studies are limited by sample size and methodologic quality issues, with a high heterogeneity across studies [62]. In a recent Cochrane systematic review on the role of probiotics for treatment of chronic constipation in children, Wallace and colleagues found that there is insufficient evidence as to whether probiotics are effective in changing the frequency of defecation or achieving global treatment success, or whether there is any difference in withdrawals due to adverse events compared with placebo [62]. There is similarly insufficient evidence to make efficacy or safety conclusions about the use of probiotics in combination with osmotic laxatives compared with laxatives alone, probiotics compared with magnesium oxide, synbiotics and paraffin compared with paraffin alone, or synbiotics compared with paraffin [62].

The potential role of probiotics, prebiotics, and synbiotics has also emerged as an attractive therapeutic strategy for modulating the gut microbiome in the management of patients with CKD, with or without constipation. Oral administration of synbiotics containing different strains across the genera *Lactobacilli*, *Bifidobacteria*, and *Streptococcus* for 6 weeks in pre-dialysis CKD patients showed significant reduction in serum uremic toxin p-cresyl sulfate in patients with CKD, but there were no significant changes in serum concentrations of indoxyl sulfate, inflammatory markers (IL-1β, IL-6, IL-10, and TNF-α), endotoxin, or eGFR [63]. The study found that synbiotics favorably modified the stool microbiome with enrichment of *Bifidobacterium* and the depletion of Ruminococcaceae [63]. The use of probiotics is also pointing toward possible beneficial effects. In a randomized, double-blind trial, there was significant decrease in serum pro-inflammatory endotoxin and cytokine levels (TNF-α, IL-5, IL-6) and a rise in serum IL-10 levels, as well as the preservation of residual kidney function after 6 months of treatment with a probiotic in patients on PD [64]. Numerous other studies have suggested that probiotics, prebiotics, and synbiotics could potentially reduce uremic toxin concentrations [65, 66], but it is currently unclear whether such effects on biomarkers lead to any beneficial clinical outcomes, and the efficacy and safety of these supplementations have yet to be confirmed in high-quality, adequately powered, randomized control trials (RCTs).

#### Psychological and behavioral interventions

Psychological and behavioral interventions are considered an important part of clinical management in children, particularly regarding counseling families to recognize withholding behaviors [11, 12]. Interventions include scheduled toileting and support to establish a regular bowel habit, maintenance and discussion of a bowel diary, information on constipation, and use of encouragement and rewards systems for successful evacuations [11, 12]. While there is limited evidence to support the clinical effectiveness of psychological and behavioral interventions, the recommendation based on expert opinion is that any psychological and/or behavioral intervention is implemented alongside effective laxative therapy and individualized to the child’s development stage [11, 12]. It is worth emphasizing that the problem of constipation can further add to the wider psychosocial burden experienced by children with CKD, highlighting the importance of appropriate behavioral interventions in this patient group.

### Pharmacological treatment

Laxatives are the mainstay of pharmacological therapy for both the treatment of fecal disimpaction and maintenance therapy. They represent a heterogeneous group of drugs which differ in their mechanisms of action and are administered as monotherapy or in combination (Table 1).

**Table 1:** Types of laxatives

Type	Drug	Mechanism of action	Considerations in children with chronic kidney disease
Osmotic	• Lactulose • Magnesium salts • Polyethylene glycol (PEG)	Create an osmotic gradient within the intestinal lumen drawing fluid into the intestinal lumen, promoting stool softening and propulsion	• High-sodium content in PEG preparations with electrolytes • Hyperphosphatemia may be associated with PEG preparations without electrolytes • Large fluid volumes required for administration of PEG • Long-term treatment with magnesium salts may cause hypermagnesemia, hypophosphatemia, secondary hypocalcemia
Stimulant	• Bisacodyl • Glycerol • Senna • Sodium picosulfate	Act on the intestinal mucosa, increasing water and electrolyte secretion and stimulating peristaltic action	• Absence of published evidence to support its use in children • May be associated with increased likelihood of side effects including diarrhea and abdominal pain
Fecal softener	• Docusate calcium • Docusate sodium	Decrease surface tension and increase penetration of intestinal fluid into the fecal mass	• Paucity of published evidence to support its use in adults and children
Lubricant	• Liquid paraffin	Lubricating effect between the feces and the intestinal wall	• Not recommended for children who are at a risk of aspiration pneumonia • May reduce absorption of fat-soluble vitamins although the clinical relevance is uncertain
Bulk	• Bran • Ispaghula husk • Methylcellulose *• Sterculia*	Retain fluid in the stool, increasing stool weight and consistency which in turn stimulate intestinal motility	• Adequate fluid intake must be maintained to avoid intestinal obstruction
Other	• Linaclotide • Lubiprostone • Prucalopride	Act directly on different receptors and channels within the intestinal epithelium, influencing and stimulating intestinal motility and fluid secretions	• Clinical trial data do not support their use in children

#### Polyethylene glycol

Polyethylene glycol (PEG), an osmotic laxative, is a non-toxic, non-absorbed water-soluble polymer that is not metabolized by the colonic bacteria. It does not carry an electrical charge and has minimal influence on the movement of other solutes. By virtue of its osmotic action, PEG works by retaining water in the stools increasing stool volume which stimulates colon motility via neuromuscular pathways. The physiological consequence is an improved propulsive colonic transportation of the softened stools. Of all the different types of laxatives, PEG is the most frequently studied in children, underpinning the NASPGHAN and ESPGHAN joint guidance recommendation to use PEG with or without electrolytes as the first-line treatment in children for both the initial disimpaction and maintenance phases of treatment [11].

A Cochrane systematic review of 17 studies finds PEG to be moderately more effective at improving the frequency of defecation in children with chronic constipation when compared to placebo and more effective than other agents, such as lactulose and milk of magnesia [67]. However, the authors provide a cautionary note that the strength of the evidence on which the recommendation is based is limited by sparse data, clinical and statistical heterogeneity, and a high risk of bias in some studies [67]. Moreover, RCTs have in general measured short-term outcomes with follow-up of 12 weeks or less, and the long-term effectiveness of PEG has not been established [67]. The optimal dose of PEG also warrants further investigation [67]. Overall, PEG with or without electrolytes has a good safety profile, with data from observational studies supporting its long-term use in otherwise healthy children with chronic constipation [68, 69].

However, particular attention should be given to the use of PEG in the context of CKD. Most preparations of PEG contain electrolytes (Table 2); electrolytes are included to allow for an iso-osmotic absorption of water into the gut and mitigate the possibility of electrolyte imbalance and dehydration. While this is perhaps beneficial for healthy individuals [70], it can lead to a considerable sodium excess in the oligo-anuric dialysis patient. Each 13-g sachet of PEG (e.g., Movicol®, Cosmocol®) contains 187 mg (8 mmol) of sodium, equivalent to 7.8–12.5% of the KDOQI daily recommended sodium intake for children with CKD (1500–2400 mg/day). Therefore, a maintenance dose of 3 to 4 sachets/day, which is not uncommon in some patients with CKD, would contribute a significant sodium load. These PEG preparations also contain potassium with 46.6 mg (0.675 mmol) of potassium chloride in each 13-g sachet.

**Table 2:** Electrolyte contents of oral laxative preparations

Drug	Formulation	Brand	Sodium content	Potassium content	Other significant content
Polyethylene glycol 3350 with electrolytes	Powder for oral solution	Movicol® Cosmocol® Laxido®	8 mmol in each 13-g sachet	0.675 mmol in each 13-g sachet	2 mmol of bicarbonate in each 13-g sachet
Polyethylene glycol 4000 without electrolytes	Powder for oral solution	Forlax® Pegorion® PegLax®	< 1 mmol in each sachet	–	–
Lactulose	Oral solution	Duphalac®	–	–	–
Sodium picosulfate	Oral solution	Dulcolax®	< 1 mmol in each 5 mL	–	240 mg of ethanol in each 5 mL
	Powder for oral solution	Picolax® CitraFleet®	< 1 mmol in each sachet	5 mmol in each sachet	87 mmol of magnesium in each sachet
Senna	Syrup	Senokot®	–	0.1 mmol in each 5 mL	6.9 mg of ethanol in each 5 mL
	Tablet	Senokot®	–	–	–
Bisacodyl	Tablet	Dulcolax®	–	–	–
Docusate sodium	Oral solution	Docusol®	< 1 mmol in each 5 mL	–	< 1 mmol of phosphate in each 5 mL
	Capsule	DulcoEase® Dioctyl®	< 1 mmol in each capsule	–	–

Electrolyte contents may vary by brand

Moreover, reports of hyperphosphatemia have been described in children receiving long-term PEG without electrolytes, although the etiology of hyperphosphatemia in these cases remains unclear [69]. A further point to note is that while it is recommended that each 13-g sachet of PEG should be mixed with 125 mL of liquid, it is commonly mixed in much smaller volumes in children on a restricted fluid allowance. It is not known whether this adapted method of preparation affects the osmotic pressure of the PEG solution and thus the effectiveness and safety of PEG. Further trials of PEG with or without electrolytes in the setting of CKD are therefore warranted.

#### Lactulose

Lactulose is also an osmotic laxative which, as previously mentioned, has been shown to be relatively less effective than PEG for the treatment of childhood constipation in the general population [67]. Experts from the NASPGHAN and ESPGHAN have recommended lactulose as an alternative if PEG is not available [11]. However, its potential place in therapy in CKD warrants further investigation given its dual function as a virtually non-absorbable synthetic disaccharide laxative and as a prebiotic.

In the colon, lactulose is metabolized by bacterial enzymes to short-chain fatty acids as well as methane and hydrogen, with consequent lowering of the colonic pH and increasing of the osmotic pressure in the colon. This causes stimulation of peristalsis and an increase of the water content of the feces. Moreover, the effect of lowering the colonic pH may also be beneficial for improving gut dysbiosis. Tayebi-Khosroshahi et al. investigated the effect of lactulose over an 8-week period on fecal microflora in a double-blind randomized placebo-controlled trial of 32 adult patients with CKD stages 3 and 4 [71]. The team observed significant increase in fecal bifidobacterial and lactobacillus counts in patients receiving lactulose, while there was no change in the placebo group [71]. Consistent with their previous findings [72], they also showed that plasma creatinine significantly decreased after lactulose administration (3.90 ± 1.43 to 3.60 ± 1.44 mg/dL, *P* = 0.003) while an increase was observed in the placebo group (3.87 ± 2.08 to 4.11 ± 1.99 mg/dL, *P* = 0.03), although no significant difference in blood urea nitrogen concentrations was observed in their latest study [71]. More recently, the effects of lactulose on kidney function and gut microbiota were further investigated in a rat model where the results are suggestive of suppression of uremic toxin production and delaying CKD progression [73].

#### Stimulant laxatives

Based on expert opinion, the use of oral stimulant laxatives, such as senna, sodium picosulfate, and bisacodyl, may be considered as an additional or second-line treatment in the management of childhood constipation [11]. Through direct stimulation of the colonic mucosa, stimulant laxatives stimulate peristalsis but also increase fluid secretion in the colon.

Unlike PEG and bulk-forming laxatives which should be taken with a relatively large amount of water, it can be reasoned why stimulant laxatives may be preferred by some nephrologists, particularly in patients with restricted fluid allowance. However, the evidence base for these therapeutic agents is scarce; there are only a few RCTs in adults [74, 75], and no RCTs were identified in the most recent Cochrane review of childhood constipation [67]. While published evidence is limited, the two studies assessing the efficacy of oral sodium picosulfate and bisacodyl have shown that both stimulant laxatives are significantly more effective than placebo [76, 77]. In the pre-dialysis CKD setting, Nata et al. investigated the efficacy of senna plus ispaghula husk and lactulose in adult patients with constipation using a cross-over study design (14 days in each period) and reported comparable efficacy between the two groups [53].

It should be noted that the two placebo-controlled studies assessing the efficacy of oral sodium picosulfate and bisacodyl were of short duration (4 weeks), and safety concerns over their long-term effects on the enteric nervous system remain a debatable topic [78]. There are no head-to-head trials comparing stimulant laxatives and PEG, but the adverse event profile of stimulant laxatives would appear less favorable. Even with short-term use, there were significantly more reports of diarrhea and abdominal pain associated with the use of bisacodyl and sodium picosulfate as compared to placebo [76, 77].

Contrary to its name, the oral solution formulation of sodium picosulfate contains less than 1 mmol of sodium per 5 mL dose and may be preferred in terms of the electrolyte content when compared to PEG (Table 2). However, caution should be taken when using preparations containing sodium picosulfate with magnesium citrate (e.g., Picolax®, CitraFleet®) as each sachet contains 5 mmol of potassium and 87 mmol of magnesium (Table 2). These preparations are licensed for bowel preparation before radiological examination, endoscopy, or surgery, and their use for constipation is off-label. Yet, intermittent use of these preparations in individuals with refractory constipation is common practice.

Glycerol suppositories are used clinically to treat constipation in both adults and children and are generally considered as a safe and effective choice for young infants where licensed treatment options are limited. However, there do not appear to be any published objective data on the use and safety of glycerol suppository, and its place in evidence-based practice remains unclear.

#### Lubricants

Liquid paraffin (or mineral oil) works primarily as a stool lubricant, although conversion of liquid paraffin to hydroxy fatty acids in the colon is also thought to induce an osmotic effect [79]. A pooled analysis of two studies found no statistically significant difference in efficacy between PEG and liquid paraffin (mean difference = 0.35, 95% confidence interval =  − 0.24 to 0.95) [67]. Comparing liquid paraffin with lactulose, a meta‐analysis of two studies revealed a relatively large statistically significant difference (mean difference = 4.94, 95% confidence interval = 4.28 to 5.61) in the number of stools per week favoring liquid paraffin [67]. While no serious adverse events were reported in the RCTs described above [67], concerns over its safety may have limited the use of liquid paraffin [79]. Safety concerns include impaired absorption of fat-soluble vitamins and risks of granulomatous disease of the gastro-intestinal tract or of lipoid pneumonia on aspiration [79].

#### Fecal softeners

Despite a paucity of evidence of effectiveness [11], even for adults [80], docusate continues to be frequently prescribed in everyday clinical practice and is recommended by the UK NICE guidance on constipation as an add-on therapy for children if hard stools are a problems [12]. Unlike the other types of laxatives, docusate acts by lowering the surface tension and increasing penetration of intestinal fluid into the fecal mass.

#### Bulk-forming laxatives

Bulk-forming laxatives, which are fiber-based, act by retaining fluid within the stool, increasing fecal mass, and stimulating peristalsis. They include bran, ispaghula husk, *Sterculia*, psyllium, and methylcellulose, where the latter also have stool-softening properties. While the World Gastroenterology Organisation indicates that psyllium supplementation may be appropriate in the management of chronic constipation in adults [81], bulk-forming laxatives are not recommended for children, especially those on a restricted fluid intake, as adequate fluid intake must be maintained to avoid intestinal obstruction. However, fiber supplements may be necessary in some children with CKD as discussed in the earlier section of this review.

#### Lubiprostone, linaclotide, and prucalopride

Therapeutic interventions with different mechanisms of action have been developed in recent years and include lubiprostone, linaclotide, and prucalopride. Lubiprostone activates type 2 chloride channels, whereas linaclotide activates guanylate cyclase-C on the luminal surface of the intestinal epithelium and prucalopride is a selective, high-affinity 5-hydroxytryptamine receptor agonist. These drugs act directly on different receptors and channels within the intestinal epithelium, influencing and stimulating colonic motility and fluid secretions, leading to increased frequency of bowel movements as well as improvement in various constipation symptoms. They are currently only licensed for use in adults where dosage adjustment is required for prucalopride in those with GFR < 30 mL/min/1.73 m^2^. In children with refractory functional constipation, the limited clinical trial data to date do not support the effectiveness of lubiprostone, linaclotide, or prucalopride, as compared to placebo [82–84], although all three drugs were found to be well tolerated with a safety profile consistent with studies in adults.

Interestingly, lubiprostone and linaclotide may have a renoprotective effect beyond the primary intended effect of relieving constipation. Using an adenine-induced kidney failure mouse model, Mishima et al. demonstrated that lubiprostone treatment altered the microbial composition, especially the recovery of the levels of the Lactobacillaceae family and *Prevotella* genus, which were significantly reduced in mice with kidney failure [85]. Also, lubiprostone treatment decreased the plasma concentration of uremic toxins, including indoxyl sulfate and hippurate, suggesting its therapeutic potential for CKD through reducing the accumulation of uremic toxins by improving the gut microbiota and intestinal environment [85]. The potential renoprotective properties of linaclotide have also been demonstrated using the same mouse model where a reduction of plasma concentrations of gut-derived uremic toxins was observed following linaclotide treatment [86].

#### Enemas and suppositories

In addition to glycerol suppository, laxatives, including bisacodyl, docusate sodium, sodium picosulfate, and liquid paraffin, are also available as enemas and suppositories. The NASPGHAN and ESPGHAN working group recommends short-term use of enemas for the treatment of fecal impaction if PEG is not available, while the addition of enemas to the chronic use of PEG is not recommended in children with constipation [11]. The relative effectiveness and safety among the different enema or suppository preparations were not specified [11]. Similarly, the UK NICE guidance recommends the use of sodium citrate enemas only if all oral medications for disimpaction have failed [12], but these recommendations are based on a limited number of low-quality trials [67]. Compared to the enema group, the PEG group had reduced chance for successful disimpaction, but the difference was not statistically significant (risk ratio 0.85, 95% confidence internal: 0.66 to 1.09) and the use of PEG was also associated with a higher frequency of fecal incontinence and watery stools [87]. Taking into consideration that administration of suppositories and enemas can be difficult, with one study reporting greater level of distress in the enema group [88], the issue of patient acceptability is likely to have influenced the recommendations provided [11]. Most importantly, phosphate enemas (which contain 119 mmol of sodium and 5.1 g of phosphorus per 118 mL dose) should be avoided in all patients with CKD and on dialysis due to the risk of life-threatening hypernatremia and hyperphosphatemia. A warning communication was issued by the U.S. Food and Drug Administration referring to reports of severe dehydration and serious harm to the kidneys and heart with a single dose of sodium phosphate that was larger than recommended or with more than one dose in a day [89].

## Conclusion

There is accumulating evidence that constipation is independently associated with adverse clinical outcomes in patients with CKD. This new understanding of the relationship offers opportunities for therapeutic intervention, but future advances will rely on understanding the role of CKD-related gut dysbiosis which plays a central role in driving the pathogenesis of both conditions in a vicious bidirectional manner. It should be emphasized that although various therapeutic options exist and are widely used in clinical practice for constipation management in children, high-quality RCTs are lacking, particularly in children with CKD.

While the lack of evidence does not equate to evidence of no effect, well-designed clinical trials are necessary to assess the efficacy and safety of various laxatives and comparisons among them in order to deliver evidence-based management plans for our patients. The multifactorial nature of constipation along with dietary restrictions in the context of CKD presents further challenges, calling for a better understanding of the extent to which different combinations of non-pharmacological and pharmacological therapy are associated with improved patient outcomes. The risks and benefits of treatment may be dependent upon and should carefully consider clinical characteristics such as CKD stage and modality of kidney replacement therapy. In the absence of robust evidence to support the use of a specific therapeutic agent in children with CKD, an individualized approach should be considered, considering the child’s diet and fluid allowance, medication preferences, and risk of potential treatment side effects. It is time to develop CKD-specific treatment strategies for the management of constipation, as well as to evaluate the potential favorable effects of various interventions on slowing the progression of CKD and reducing cardiovascular complications.

## Key summary points

- Several features and complications of chronic kidney disease, including the uremic milieu, fluid and diet restriction, decreased physical activity, and concomitant medications, predispose to constipation.
- Emerging evidence suggests a possible bidirectional relationship between constipation and CKD, potentially mediated via gut dysbiosis in a vicious circle.
- The available treatments for constipation have different mechanisms of action, but the evidence base is limited in the CKD population.
- Prescribers should exercise care when prescribing laxatives in the setting of CKD as some preparations contain significant amounts of electrolytes, including sodium and potassium, as excipients.

## Multiple-choice questions

1. Factors contributing to constipation in children with CKD include:
  - Reduced fluid intake as kidney function deteriorates
  - Toileting behaviors
  - Reduction in physical activity
  - Medications used in the management of CKD
  - All of the above
2. The understanding of gut dysbiosis is important in CKD because:
  - There is evidence that it directly leads to disease progression
  - Evidence suggests some increased cardiovascular risk
  - Dysregulation leads to worsening uremia
  - b and c
  - All of the above
3. Children on dialysis who are constipated, can have hyperkalemia. This can be due to:
  - CKD leads to an intracellular accumulation of potassium
  - Constipation reduces intestinal transit time, leading to increased potassium absorption
  - In healthy individuals up to 80% of potassium is excreted through the gastrointestinal tract
  - All laxatives contain potassium salts
  - All of the above
4. Which of these laxatives has the highest sodium content?
  - Isotonic Polyethylene Glycol (PEG)
  - Ispaghula husk
  - Docusate sodium
  - Bisacodyl
  - Sodium picosulphate
5. Children with CKD requiring pharmacological management of constipation:
  - Are more often on peritoneal dialysis than hemodialysis
  - Often require one agent for optimal management
  - Require care to be taken when prescribing multiple agents as they may contain sodium and potassium salts
  - Require large volumes of laxatives for optimal management
  - Require large volumes of fluid for optimal management

### Supplementary Information

Below is the link to the electronic supplementary material.

Supplementary file1 (DOCX 17 KB)

## Data Availability

Not applicable.
