# Supplementary material for: Gutted: constipation in children with chronic kidney disease and on dialysis
Source: Pediatr Nephrol. 2023 Jan 9;38(11):3581–96. doi: 10.1007/s00467-022-05849-y (PMC10514126; doi:10.1007/s00467-022-05849-y)
Supplement: Supplementary file 1 — Supplementary file1 (DOCX 17 KB) [file 467_2022_5849_MOESM1_ESM.docx]

**Supplementary Table 1: Rome criteria IV - Diagnostic criteria for functional constipation [11]**

| **Infants up to 4 years of age** |
| --- |
| Must include 1 month of at least 2 of the following:   - - - - 2 or fewer defecations per week - History of excessive stool retention - History of painful or hard bowel movements - History of large-diameter stools - Presence of a large fecal mass in the rectum |
| In toilet-trained children, the following additional criteria may be used:   - At least 1 episode/week of incontinence after the acquisition of toileting skills - History of large-diameter stools that may obstruct the toilet |
| **Children of a developmental age of at least 4 years** |
| Must include 2 or more of the following occurring at least once per week for a minimum of 1 month with insufficient criteria for a diagnosis of irritable bowel syndrome:   - 2 or fewer defecations in the toilet per week - At least 1 episode of fecal incontinence per week - History of retentive posturing or excessive volitional stool retention - History of painful or hard bowel movements - Presence of a large fecal mass in the rectum - History of large diameter stools that can obstruct the toilet |
| After appropriate evaluation, the symptoms cannot be fully explained by another medical condition. |

**Supplementary Table 2: Bristol Stool Form Scale [15]**

| Type 1 | Separate hard lumps, like nuts (hard to pass) |
| --- | --- |
| Type 2 | Sausage shaped but lumpy |
| Type 3 | Like a sausage but with cracks on the surface |
| Type 4 | Like a sausage or snake, smooth and soft |
| Type 5 | Soft bloc with clear cut edges |
| Type 6 | Fluffy pieces with ragged edges, a mushy stool |
| Type 7 | Water, no slid pieces, entirely liquid |
